# Crystal Structure of Mannose Specific IIA Subunit of Phosphotransferase System from *Streptococcus pneumoniae*

**DOI:** 10.3390/molecules25204633

**Published:** 2020-10-12

**Authors:** Malgorzata Magoch, Przemyslaw Nogly, Przemyslaw Grudnik, Pikyee Ma, Bozena Boczkus, Ana Rute Neves, Margarida Archer, Grzegorz Dubin

**Affiliations:** 1 Malopolska Centre of Biotechnology, Jagiellonian University, 30-387 Krakow, Poland; m.magoch@gmail.com (M.M.); przemyslaw.grudnik@uj.edu.pl (P.G.); b.boczkus@gmail.com (B.B.); 2 Faculty of Biochemistry, Biophysics and Biotechnology, Jagiellonian University, 30-387 Krakow, Poland; 3 Instituto de Tecnologia Quimica e Biologica, Universidade Nova de Lisboa, ITQB NOVA, 2780-157 Oeiras, Portugal; przemyslaw.nogly@mol.biol.ethz.ch (P.N.); pikyeema@gmail.com (P.M.); 4 Institute of Molecular Biology and Biophysics, Department of Biology, ETH Zürich, Wolfgang-Pauli-Strasse 27, 8093 Zürich, Switzerland; 5 Laboratory of Biomolecular Research, Paul Scherrer Institute, 5232 Villigen PSI, Switzerland; 6 Bacterial Physiology, R&D, Chr. Hansen A/S, Bøge Allé 10-12, 2970 Hørsholm, Denmark; DKRUNS@chr-hansen.com

**Keywords:** *Streptococcus pneumoniae*, phosphotransferase system, IIA subunit, mannose PTS, sugar transport, X-ray crystallography

## Abstract

*Streptococcus pneumoniae* is a frequent bacterial pathogen of the human respiratory tract causing pneumonia, meningitis and sepsis, a serious healthcare burden in all age groups. *S. pneumoniae* lacks complete respiratory chain and relies on carbohydrate fermentation for energy generation. One of the essential components for this includes the mannose phosphotransferase system (Man-PTS), which plays a central role in glucose transport and exhibits a broad specificity for a range of hexoses. Importantly, Man-PTS is involved in the global regulation of gene expression for virulence determinants. We herein report the three-dimensional structure of the EIIA domain of *S. pneumoniae* mannose phosphotransferase system (SpEIIA-Man). Our structure shows a dimeric arrangement of EIIA and reveals a detailed molecular description of the active site. Since PTS transporters are exclusively present in microbes and sugar transporters have already been suggested as valid targets for antistreptococcal antibiotics, our work sets foundation for the future development of antimicrobial strategies against *Streptococcus pneumoniae*.

## 1. Introduction

*Streptococcus pneumoniae***,** a natural inhabitant of human nasopharynx, is capable of progressing to sterile sites in the human body, causing serious diseases including pneumonia, meningitis and sepsis [1]. Worldwide, *S. pneumoniae* accounts for more deaths than any other bacterium [2]. According to the World Health Organization (WHO), streptococcal infections are responsible for about 1 million deaths per year in children under 5 years of age. High rate of resistance acquisition to traditional antibiotics and the re-emergence of non-vaccine type strains forebode increasing healthcare threat in the forthcoming years. Therefore, the development of new therapeutic and preventive strategies is required, which calls for better understanding of streptococcal pathogenesis.

**Figure 1:**
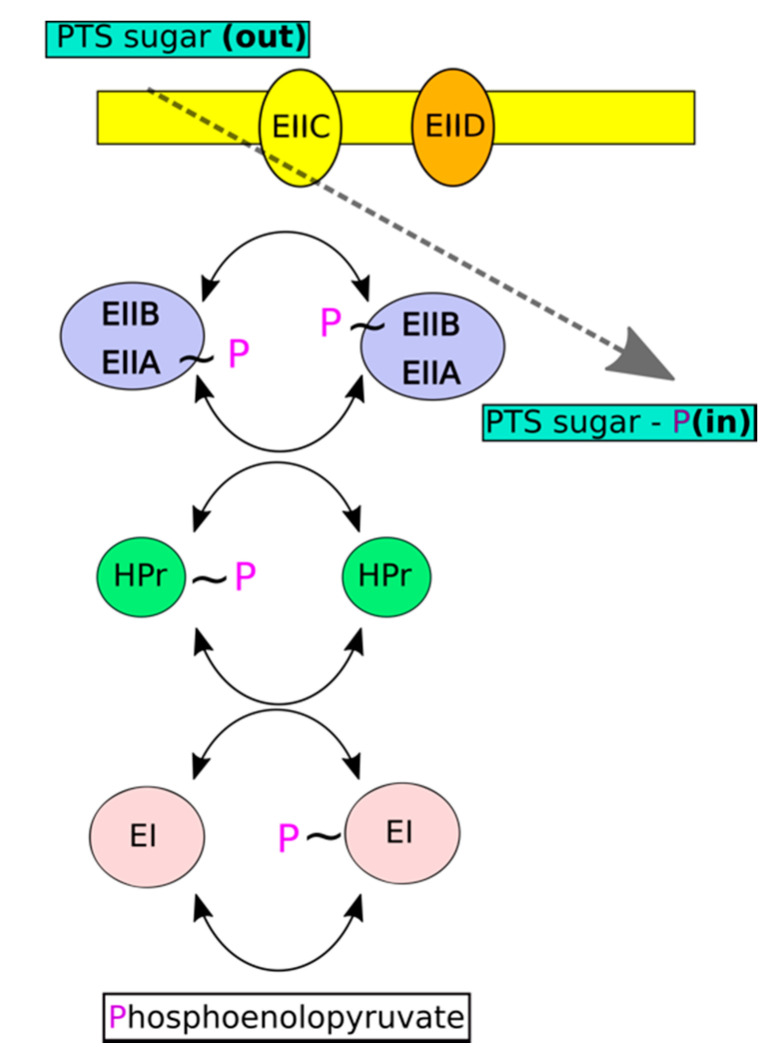
Carbohydrate transport and phosphorylation in phosphotransferase system (PTS) exemplified by mannose-PTS. A membrane embedded sugar specific permease (Enzyme II; EII; consists of several subunits designated with capital roman letters) transports sugars across the membrane with simultaneous phosphorylation. Permease regeneration is driven by Enzyme I (EI)/HPr system common to all PTSs. EI uses phophoenolopyruvate for autocatalytic phosphorylation. Phosphorylated-EI transfers the phosphoryl group at histidine containing phosphocarrier (HPr) which, in turn, donates the phosphoryl to EII.

The phosphoenolpyruvate transferase system (PTS) constitutes the major uptake pathway for various carbohydrates in Gram-positive and Gram-negative bacteria. Unlike other transport systems, PTS utilizes phosphoenolpyruvate (PEP) rather than ATP as an energy source (phosphate donor). A membrane embedded permease (EII; enzyme II) is the major functional component of each sugar specific PTS system. The permease transports sugars across the membrane with simultaneous phosphorylation to energetically drive the process [3,4]. Two cytoplasmic components ensure permease regeneration and are common to all PTS systems. Enzyme I (EI) uses phosphoenolopyruvate for autocatalytic phosphorylation and subsequently transfers the phosphonyl group to His15 of histidine-containing phosphocarrier (HPr). Phosphorylated HPr donates the phosphoryl group to EII (Figure 1).

Permeases (EIIs) subdivide into four major families according to substrate selectivity—glucose-sucrose, mannitol-fructose, mannose-sorbose, and lactose-cellobiose specific. These families share no sequence or structural similarity to one another; however, they have similar functional organization [5,6]. All permeases comprise two hydrophilic components (EIIA and EIIB) responsible for transfer of the phosphoryl group at the incoming carbohydrate and transmembrane sugar permease domain—EIIC (in some cases the permease contains additional EIID component) responsible for sugar translocation. In different EIIs, the above components may exist as distinct subunits of a noncovalent complex or as domains covalently linked in various orders [4,7]. 

The energy metabolism of *S. pneumoniae* relies on fermentation of dietary sugars [8]. Streptococcal genome encodes numerous PTS systems that provide an exceptionally high capacity for using different sugars, thus conferring adaptation to diverse niches [9]. Beyond the primary role in sugar transport, PTS systems may also participate in other important physiological processes—for example, the *Streptococcus mutans* strain, which lacks EIIAB-Man (ΔmanL), was unable to form biofilms in the presence of glucose and showed reduced competency and reduced acid tolerance [10]. PTS systems have also been implicated in chemotaxis by modulating the autophosphorylation of a central regulator of bacterial chemotaxis—CheA—which then transfers the phosphate group to CheY. Phosphorylated CheY modulates the rotational preferences (clockwise versus counterclockwise) of flagellar motors [11,12]. In vitro experiments have shown that unphosphorylated EI interacts with CheA and inhibits the autokinase activity of CheA by up to 10-fold [4]. Importantly, PTS play a major role in carbon catabolic repression (CCR), a phenomenon of hierarchical utilization of carbon sources. CCR is accomplished through the downregulation of expression of genes involved in catabolism of less favorable carbon sources, as long as the preferred sugars are present in sufficient amounts [4]. CCR is primarily regulated by a transcription factor catabolite control protein A (CcpA), which binds to catabolite-responsive elements (cre) in promoter regions [13]. The link to PTS is provided by the fact that affinity of CcpA for *cre* is enhanced by interaction with phosphorylated HPr [13,14]. By sensing the phosphorylation level of PTS, CcpA not only regulates operons involved in sugar utilization and sugar preference [4], but also regulates the expression of virulence related genes. For example, a gene encoding beta-galactosidase BgaA, which is essential for pathogenesis, has upstream *cre* elements [14]. BgaA modulates pathogen adherence to human epithelial cells by the destruction of host-cell polysaccharides [15].

Glucose (Glc) is the preferred sugar for the majority of fast growing, low-GC Gram-positive species and PTS usually constitutes the major transporter [16]. The glucose uptake pathway has not been fully characterized in *S. pneumoniae*, but Man-PTS, encoded within *manLMN* operon, constitutes an important entry route and an element of CCR [17,18]. Glucose level differs significantly between nasopharynx (5–40 μM) and blood (5 mM) and it has been suggested that niche specific virulence factors are regulated by Glc-dependent CCR. This hypothesis is supported by the fact that CcpA inactivation resulted in the decreased virulence of *S. pneumoniae* in mouse models of bacteremia and pneumonia [19].

Because PTSs are central to energy metabolism and unique to bacteria, the system components have been suggested as suitable drug targets [20]. Interestingly, nature has already provided a validation of this concept—recent studies have demonstrated Man-PTS as a major receptor of class II bacteriocins (natural bactericidal peptides) [21,22]. So far, a number of three-dimensional structures of cytoplasmic components of the mannose-PTS have been determined both by X-ray and NMR methods. Crystal structures encompassing the isolated domains from a variety of microorganisms are available for EIIA (see Section 3.3 for more details) and EIIB [9,23,24]. Complex structures of EIIA-EIIB and Hrp-EIIA from *E. coli* have also been solved using the NMR method [6,25].

Here, we report a high-resolution crystal structure of the Man-PTS EIIA domain from *S. pneumoniae* (SpEIIA-Man) providing a structural understanding of sugar transport in this important human pathogen. The detailed molecular description of the active site of EIIA provided in this study may facilitate the future rational design of novel antistreptococcal drugs.

## 2. Results and Discussion

### 2.1. Expression and Crystallization of EII Components of S. pneumoniae Man-PTS

**Figure 2:**
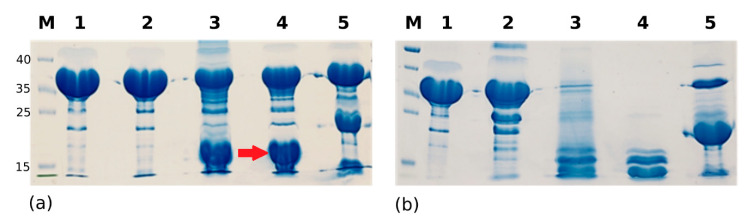
Limited proteolysis of SpEIIAB-Man: (**a**) 10% SDS-PAGE of SpEIIAB-Man after incubation with proteases at 1:1000 molar ratio: lane 1—untreated protein (control), treated with: lane 2—chymotrypsin, lane 3—papain, lane 4—subtilisin (with EIIA domain indicated with red arrow), lane 5—trypsin. (**b**) Same as panel (**a**), but with proteases at 1:100 molar ratio.

The EII of streptococcal Man-PTS contains phosphotransfer domains (SpEIIA-Man and SpEIIB-Man) and permease domains (SpEIIC-Man and SpEIID-Man). To cast light on sugar-independent reactions, we cloned and expressed SpEIIAB-Man in *E. coli* (1–329, 35 kDa). Crystallization screening was performed at room temperature and the first crystals appeared after approximately 1 month. These crystals diffracted poorly, up to ~12 Å, and optimization efforts to improve crystal diffraction quality failed. Relatively long crystallization time suggested possible processing of the initial construct. Limited proteolysis of SpEIIAB-Man was tested with several proteases at different concentrations for 30 min. SDS-PAGE analysis indicated similar bands for the control protein (no protease added) and upon incubation with chymotrypsin at 1:1000 molar ratio, indicating that this protease has no effect on our protein construct. In contrast, an additional broad band is observed around 15 kDa when papain and subtilisin are tested, which band corresponds to SpEIIA-Man. Two bands appear around 20 and 12 kDa after trypsin digestion (Figure 2a). We sequenced these bands (after trypsin), and the higher MW band corresponded to SpEIIA-Man extended by a plasmid-derived sequence and the lower band matched a fragment of SpEIIB-Man. When the proteases were used at a higher concentration (1:100) (Figure 2b), still no significant effect was observed for chymotrypsin, while other proteases significantly degraded the sample, with two more distinct bands ~15 kDa for papain and subtilisin, which could possibly correspond to SpEIIA-Man and SpEIIB-Man (sub)domains. Subtilisin at trace amounts was selected for in situ reaction in crystallization experiments. Well diffracting crystals were obtained within two days using PEG 8000 as a precipitant and no further optimization was required. 

### 2.2. Overall Crystal Structure of SpEIIA-Man

**Figure 3:**
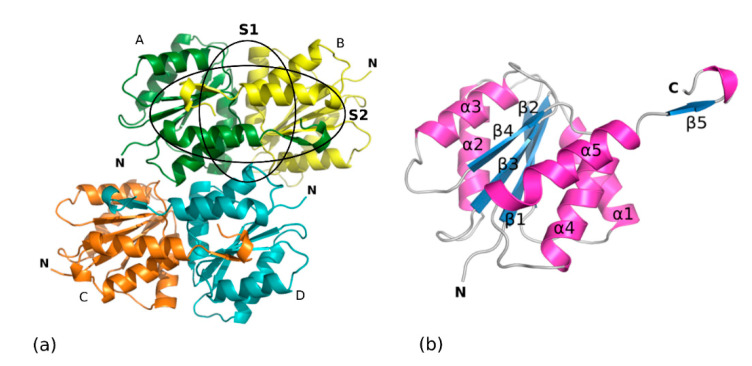
Cartoon representation of EIIA domain of mannose phosphotransferase system (SpEIIA-Man) from *Streptococcus pneumoniae* (PDB code: 6FMG). (**a**) Homotetramer present in the asymmetric unit. First dimer is colored in green and yellow and the second one is colored orange and blue. (**b**) Monomer with secondary elements numbered.

The crystals grown upon in situ proteolysis diffracted to 1.8 Å resolution and belonged to the hexagonal space group P6_3_ with unit cell parameters of a = b = 138.53 Å and c = 69.77 Å (α = β = 90°, γ = 120°). SpEIIAB-Man consists of EIIA and EIIB domains connected by a linker, which is likely solvent accessible and more prone to proteolysis. We hypothesized that the crystals could comprise only one of the domains due to the linker proteolytic cleavage. If four molecules with approximately half the molecular weight of SpEIIAB-Man are packed in the crystal asymmetric unit, the calculated V_M_ is 3.2 Å^3^/Da with 61% of estimated solvent content, which are within reasonable values [26]. Then, molecular replacement trials were performed using search models from homologous EIIA and EIIB structures from different sources, and the structure was solved using the EIIA model generated by Automatic Molecular Replacement Pipeline MoRDa [27], based on a structure of *E. coli* EIIA (EcEIIA-Man). The electron density maps are of good quality and are generally well defined, except for loops comprising residues (Pro38-Asn39-Glu40 and Glu83-Asn84-Pro85-Glu86-Arg87) and the C-terminal Pro139. The structure was refined to a final R_work_ value of 17.02% and R_free_ of 19.65% (Table 1). The final model contains four SpEIIA-Man molecules arranged as two SpEIIA-Man dimers (Figure 3a) and comprises a total of 556 amino-acid residues (139 for each monomer) and 462 water molecules.

Much of the evidence suggests that SpEIIA-Man dimer is the functional arrangement of the protein. Dimers burry an extensive surface area and are stabilized by significant hydrophobic and hydrogen bond interactions. Analysis by PISA server [28] suggests a physiological role for the dimer. The three-dimensional structures of EIIA-Man-HPr and EIIAB-Man complexes from *E*. *coli* [6,25] and the isolated domains of EIIA-Man from other organisms, have also been reported in dimeric arrangements [9,29,30,31]. Most significantly, the residues of both protomers contribute to the active site of EIIA as evidenced by the structure of the complex of Hrp-EIIA [29].

The overall structure of SpEIIA-Man monomer consists of a central β-sheet composed by four parallel β-strands (β1–β4) surrounded by two (α2, α3) and three (α1, α4 and α5) α-helices packed on either face of the β-sheet. The first four parallel strands (β1 to β4) alternate with α-helices (α1 to α4) and all crossovers are right-handed. In turn, the fifth helix (α5) forms a helical hairpin with helix α4 and the β5-strand sticks out of the globular fold (Figure 3b). Interestingly, this fifth short β-strand interacts with the adjacent molecule of the crystallographic dimer and runs anti-parallel to its central β-sheet, as depicted in (Figure 3a).

**Figure 4:**
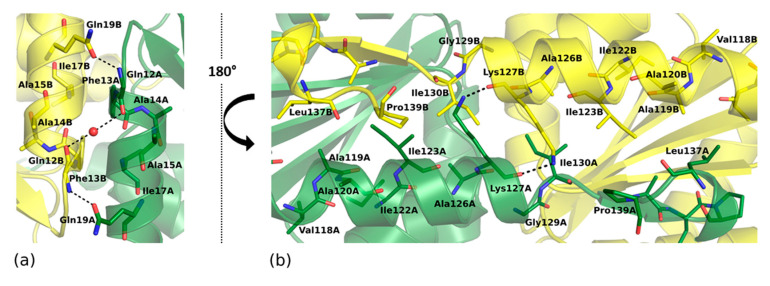
Dimeric interface of SpEIIA-Man. Secondary structure elements of protomers are represented in yellow and green. Carbon atoms are colored according to their polypeptide chain, nitrogen in blue, oxygen in red, and water molecules are depicted as red spheres (**a**) Amino acids involved in the α1–α1′ interface (S1 subsite) and (**b**) Amino acids involved α5–β5′ and α5′–β5 interfaces (S2 subsite).

SpEIIA-Man dimer buries an extensive surface area of 1839 Å^2^ which accounts for 23% of the accessible surface area of each monomer according to PISA [28]. The A–B dimer interface consists of two major subsites, both of which include mainly hydrophobic contacts. The first subsite (S1 in Figure 3a and Figure 4a) is formed by amino acid residues of α1 helices and α1/β1 loops from each monomer that establish extensive hydrophobic contacts contributed by Phe13, Ala14, Ala15, Ile17 and also comprise hydrogen bonds between Gln12A-Gln19B, Gln19A-Phe13B and Ala14A-Ala14B (mediated by a water molecule). The second subsite is formed by residues from α5 helix and β5 strand of adjacent monomers (S2 in Figure 3a and Figure 4b) that contribute a number of hydrophobic interactions. In addition, the amine sidechain of Lys127 is H-bonded to the carbonyl of Lys127 and contributes to dimer stabilization. Further contacts are provided by β-sheet backbone interactions of β5-strand flanking the β1–β4 sheet of the adjacent protomer. 

### 2.3. Structure Conservation among EIIA Components

**Figure 5:**
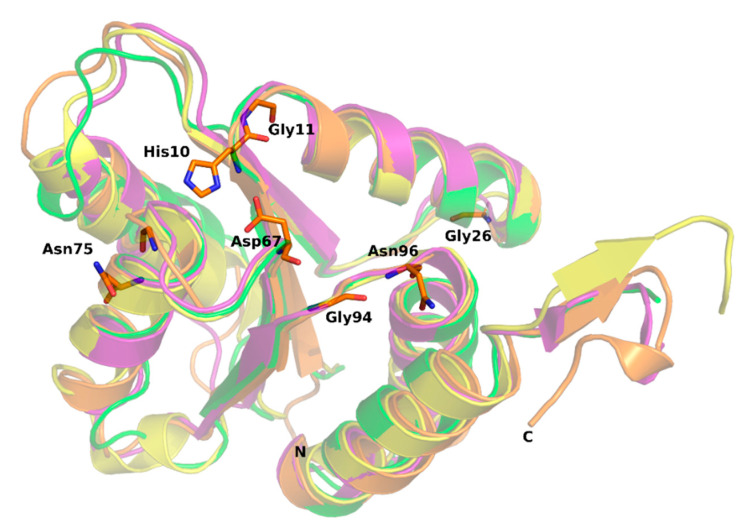
Overlay of reported X-ray structures of EIIA subunits from mannose transporters; for sake of clarity only 4 highest scoring hits out of 8 structures are displayed: 6FMG—*S. pneumoniae* (this study, orange), 4TKZ—*S. agalactiae* (purple), 3IPR—*E. faecalis* (yellow) and 1PDO—*E. coli* (green). The conserved residues are shown in the stick model (based on structure of SpEIIA-Man determined in this study).

**Figure 6:**
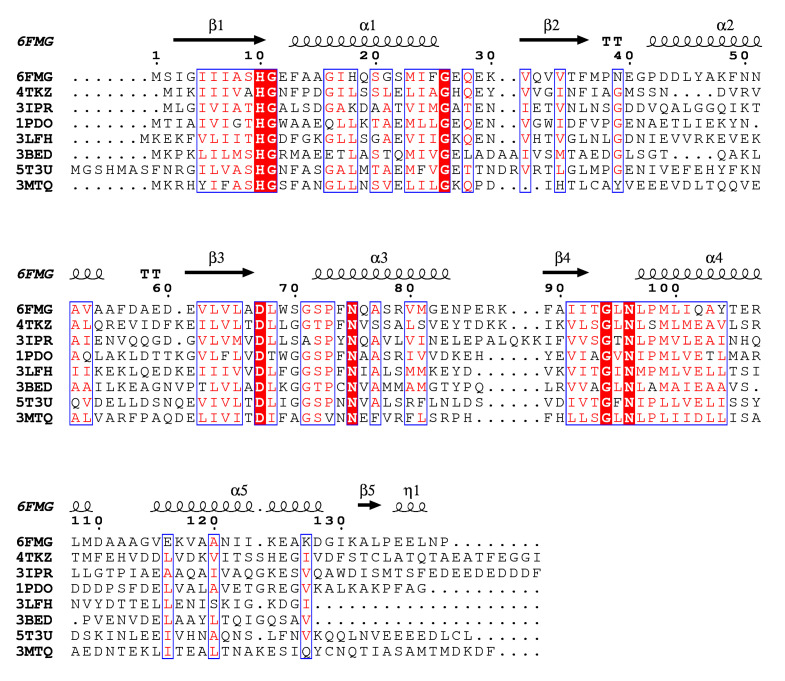
Amino acid sequence alignment of EIIA-Man domains of mannose transport proteins, for which structures have been reported. PDB codes and sources of EIIA subunits are as follows: 6FMG—*Streptococcus pneumoniae* (structure characterized in this study), 4TKZ—*Streptococcus agalactiae*, 3IPR—*Enterococcus faecalis*, 1PDO—*Escherichia* coli, 3LFH—*Thermoanaerobacter tengcongensis*, 3BED—*Enterococcus faecalis*, 5T3U—*Streptococcus pneumoniae,* 3MTQ—*Klebsiella pneumoniae*. Strictly conserved residues are highlighted by red background while homologous residues are depicted in red. Secondary structure elements are indicated above the alignment. The figure was prepared with ENDscript [33] which uses DSSP to identify the secondary structure elements [34].

Mannose transporter of *S. pneumoniae* is built of EIIA, EIIB, EIIC and EIID subunits, which share no sequence homology with other PTS families. Remarkably, among PTS permeases only the mannose family comprises the EIID domain of yet unknown function. Moreover, *S. pneumoniae* EIIB-Man uses histidine to accept the phosphoryl group from EIIA, while EIIBs from other families use cysteine instead [4]. The three-dimensional structures of EIIA subunits from mannose PTS are available for *Streptococcus agalactiae* (PDB:4TKZ), *Enterococcus faecalis* (PDB:3IPR), *Escherichia coli* (PDB:1PDO), *Thermoanaerobacter tengcongensis* (PDB:3LFH), *Enterococcus faecalis* (PDB:3BED) and *Klebsiella pneumoniae* (PDB:3MTQ). In addition, *S. pneumoniae* fucose transporter, a protein distinct from SpEIIA-Man, but belonging to the same mannose family has been reported (PDB:5T3U) [9]. Interestingly, despite the relatively low amino acid sequence identity among SpEIIA-Man (from 26 to 36%) (Figure 5), their overall fold is quite conserved and similar to the herein characterized SpEIIA-Man structure (Figure 6). A DALI [32] search using 6FMG shows a root mean square deviation (RMSD) in the range of 1.5–2.2 Å over 139 residues and Z-scores between 21.6–17.4 among the available structures containing a SpEIIA-Man domain (Table 2). Such structural homology allows us to infer about functional features of SpEIIA-Man. Seven residues are strictly conserved among known EIIA-Mans, and thus likely to have important functional or structural role (Figure 5). Previous studies indicated that His10 (SpEIIA-Man numbering) is the phosphate acceptor/donor, which orientation is stabilized by Asp67. Gly71 lies in a hydrophobic pocket that accommodates the side-chain of His10 from an adjacent protomer. Gly11 locates at the beta strand β1 and Gly26 at helix α1, which are followed by loops. These two glycines are proposed to play a structural role, as their side chains (hydrogen atoms) cause no steric hindrance and may contribute to loop flexibility.

Asn75, Gly94 and Asn96 are likely to contribute to specific interactions with HPr, as suggested by structural analysis of *E. coli* binary complex of HPr with EcEIIA-Man [29]. Additionally, the hydroxyl group of Ser72 (Ser/Thr in other EIIA-Man) is involved in phosphate positioning during phosphotransfer (see more details below).

### 2.4. Active Site of SpEIIA-Man

**Figure 7:**
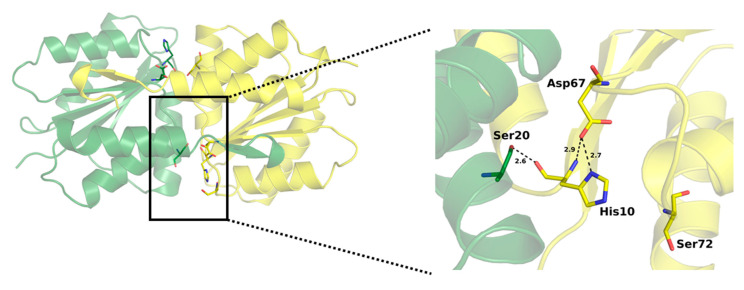
Active site SpEIIA-Man. Catalytic residues are shown in stick representation: His10, Asp67, Ser72 (see text for the description of the catalytic process).

**Figure 8:**
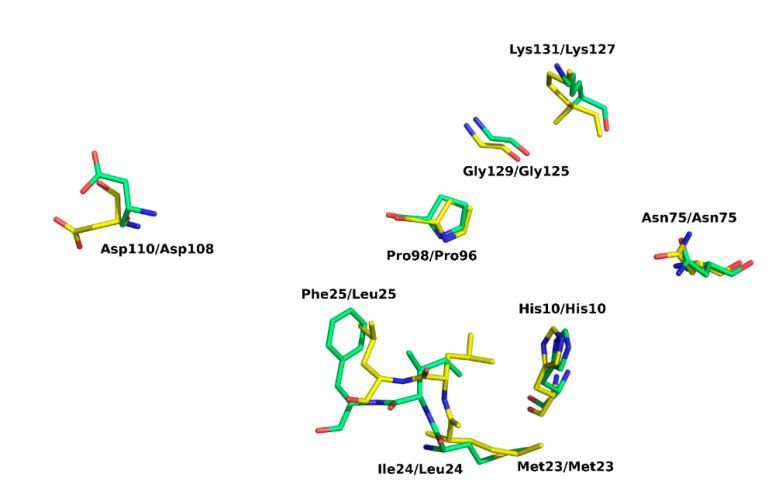
Comparison of the active site architecture of SpEIIA-Man (green) and EcEIIA-Man (yellow). Key amino acids are shown in a stick representation. Side chain nitrogens and oxygens are displayed in blue and red, respectively.

Comparative analysis of our structure in the light of earlier data allows a comprehensive description of the active site. NMR studies of *E. coli* EIIA-Man-HPr and EIIAB-Man complexes provided information on the interaction surfaces on EIIA-Man and molecular bases of phosphate moiety transfer [6,25]. SpEIIA-Man has a comparable fold and monomer organization to EcEIIA-Man. It follows that the active sites are very similar in those two proteins. A characteristic hydrophobic pocket is found in *E. coli* EIIA-Man adjacent to active site His10. The pocket is formed by residues Phe36, Met23, Leu24, Leu25, and Met103. We show that, in *S. pneumoniae*, a corresponding pocket is formed by the replacement of residues: Leu24 to Ile24, Leu25 to Phe25 and Met103 to Thr105. The N-ε2 amide of the imidazole ring of His10 (*E. coli*) is facing the protein interior and is in hydrogen bonding distance (2.7 Å) to the completely buried Asp67 that stems from loop 3/C. Comparable arrangement and hydrogen bond compatible distance is observed in our structure (Figure 7). In *E. coli* the active site imidazole is surrounded by apolar residues Gly71A, Ser72A, Pro73A and Phe36A, and Met23B and Leu24B from adjacent subunit. The entire site is very closely resembled in SpEIIA-Man, with only a single sidechain substitution at positon 24 where isoluecine is found in place of leucine. By further exploring the structural analogies here we propose the following sequence of events: the SpEIIA-Man surface that interacts with Hpr originates from both monomers (subunit A and B) and encompasses the area around the essential His10 of subunit A where the surface exhibits a shallow groove, complement to convex surface of Hpr. This interaction between SpEIIA-Man and HPr involves mainly helix–helix contact. In the case of *E. coli* EIIA-Man, there are extensive interactions between Met23, Leu24, Leu25, Pro96 and Gly125 of subunit B and Ala20, Val23, Leu47, and Phe48 of HPr. It corresponds to Met23B, Ile24B, Phe25B, Pro98B and Gly129B in *S. pneumoniae*, where Met23B, Pro96 (98) B and Gly125 (129) B are conserved among these two organisms and probably play an essential role in Hpr recognition. There are also a number of potential electrostatic and hydrogen-bonding interactions that might contribute to the specificity of the interaction as well as to set the correct orientation of HPr and EIIA-Man. Comparison between EcEIIA-Man with SpEIIA-Man reveals the conservation of the following residues: Asn75, Asp108 and Lys127 (*E. coli*) and their equivalents in *S. pneumoniae*: Asn75, Asp110 and Lys131 (Figure 8).

Prior to phosphorylation, His10A is stabilized in a solvent exposed orientation by a hydrogen bond with the carboxyl of Asp67A, which stems from loop β3/α3 and is completely buried inside the active site pocket (Figure 7). The orientation of the imidazole ring is further stabilized within the hydrophobic cavity, comprising Gly71A, Ser72A, Pro73A (of loop β3/α3), Phe36A (of loop β2/α2), Leu68A (loop β3/α3) as well as Met23B and Ile24B of the adjacent subunit of SpEIIA-Man. Asp67A not only orients the His10A imidazole ring in a conformation accessible for the phosphoryl transfer, but more importantly, it facilitates its nucleophilic attack on the phosphorus of Hpr (P-HPR) by acting as a general base and increasing the basic strength of the imidazole ring of His10A [29]. The His-Asp pair is consistent in all homologues of EIIA-Man. This system is comparable to serine proteases where a similar His-Asp pair is part of the catalytic triad. In serine proteases, a strong, low-barrier hydrogen bond (LBHB) with aspartic acid increases the reactivity of active site histidine as a general base [35]. The short length of mentioned bonds in serine proteases (~2.7 Å) [36] is comparable to that observed in our structure between Asp67 and His10, suggesting the activating role of Asp67 on His10.

Phosphoryl transfer occurs from the N-δ1 atom of His15 of HPr to the N-ε2 atom of His10A of EIIA-Man by forming an associative transition state involving a pentacoordinate phosphoryl group in a trigonal bipyramidal geometry. The phosphoryl group is H-bonded to the hydroxyl group of Thr16 and the backbone amides of Thr16 and Arg17 on the HPr side and to the hydroxyl group of Ser72A and the backbone amide of Ser72A on the EIIA-Man (Ser/Thr in homologous structures) side localized at the N-terminus of helix C in EIIA. This interaction is important since it facilitates the phosphonyl transfer. It has been demonstrated that the Ser72Cys mutant of the EcEIIA-Man retains less than 6% of wild type activity [37]. This residue also appears to stabilize the dimer. Further in the cascade, the phospho-histidine intermediate transfers the phosphonyl to His175 of EIIB-Man.

## 3. Materials and Methods

### 3.1. Cloning, Expression and Purification of SpEIIAB-Man

Gene encoding the full-length SpEIIAB-Man (Uniprot: A0A062WNX6, residues 1–329) was codon optimized for expression in *E. coli*, synthesized (Genescript) and cloned into the pETM-11 plasmid using NcoI/SacI restriction sites. *E. coli* BL21 Star (DE3) (Invitrogen) were transformed with pETM-11-SpEIIAB-Man and cultured in Terrific Broth (Bioshop) at 37 °C until OD_600_ reached ~0.6. Gene expression was induced with isopropyl β-D-1-thiogalactopyranoside (IPTG; 0.5 mM) and the cells were cultured for additional 3 h at 37 °C. Cells were harvested by centrifugation (5000× *g* for 15 min at 4 °C) and the pellet was stored at −20 °C.

Pellets were thawed, resuspended in 50 mM Tris pH 7.5 containing 200 mM NaCl, 20 mM imidazole, 0.5 mM EDTA, protease inhibitor cocktail (cOmplate, Roche) and BugBuster (Novagen) supplement. The lysate was incubated at 20 °C for 20 min and cleared by centrifugation (16,000× *g* for 20 min at 4 °C). The protein of interest was purified on Chelating–Sepharose (HisTrap HP, Amersham Biosciences) equilibrated with 50 mM Tris pH 7.5, 500 mM NaCl, 20 mM imidazole. The column was washed thoroughly using the same buffer and the fusion protein was eluted with 50 mM Tris pH 7.5 containing 50 mM NaCl and 500 mM imidazole. SpEIIAB-Man was further purified by size-exclusion chromatography (Superdex 75, GE Healthcare) in 10 mM Tris pH 8.0 containing 100 mM NaCl. Fractions containing the protein of interest were identified by SDS-PAGE.

### 3.2. Limited Proteolysis

Trypsin, chymotrypsin, subtilisin and papain (Jena Bioscience) were incubated at 1:100 and 1:1000 molar ratios with SpEIIAB-Man at room temperature for 30 min and the progress of proteolysis was monitored by SDS-PAGE (Figure 2).

### 3.3. Crystalization of SpEIIAB-Man

SpEIIAB-Man was concentrated to ~40 mg/mL and used for crystallization screening directly or after supplementing with trace subtilisin (1:1000) immediately prior to crystallization. Crystallization screens were performed using sitting-drop vapor diffusion method and commercially available crystallization kits (Structure Screen 1+2 and NR-LBD from Molecular Dimensions). 1 µL drops of protein solution were mixed with an equal volume of the reservoir solution and incubated at 20 °C. Initial crystals appeared after ca. one month in 0.1 M Tris pH 8.5 containing 20% Ethanol (no protease) and in 0.1 M sodium cacodylate pH 6.5 containing 0.2 M magnesium acetate tetrahydrate and 20% *w/v* PEG 8000 (protease supplemented). Diffraction data were measured directly from crystals obtained in screening.

### 3.4. Data Collection and Crystal Structure Solution

Crystals were cryo-protected in reservoir solution supplemented with 20% glycerol and flash-cooled in liquid nitrogen. X-ray diffraction data were collected on BL14.2 beamline operated by the Helmholtz-Zentrum Berlin (HZB) at the BESSY II electron storage ring (Berlin-Adlershof, Germany). Data obtained from the crystal of in situ subtilisin processed SpEIIAB-Man were indexed and integrated using XDS package with XDSAPP2.0 graphical user interface [38] and scaled using SCALA [39,40]. 5% of reflections was set aside to calculate R_free_ and monitor the refinement process. The initial phases were obtained by molecular replacement with Phaser [41] using the structure of mannose IIA subunit from *E. coli* (EcEIIA-Man; 36% of amino acid identity, PDB ID: 1PDO) as a search model. The initial model was refined using alternated cycles of model building with Coot [42] and crystallographic refinement with Refmac5 [43] contained in CCP4 package until convergence was achieved with R_work_/R_free_ values of 17.02/19.65%, respectively (Table 1). Structure coordinates were deposited in Protein Data Bank with the accession code 6FMG. Data collection and refinement statistics are summarized in Table 1.

## 4. Conclusions

This study provides the structure elucidation of EIIA subunit of mannose transferase system from *Streptococcus pneumoniae* at atomic level. Comparison with available structures allows inference about the events leading to phosphoryl moiety transfer from a common donor Hpr to SpEIIA and later to SpEIIB, which is the primary achievement of this study. Elucidation of the molecular architecture of the catalytic pocket comprising His10, a structural key element to the activity of SpEIIA offers the opportunity to target this feature with rationally designed small molecule inhibitors, providing a possible future strategy to fight streptococcal infections.

## Figures and Tables

**Table 1:** Crystallographic Data Collection and Refinement Statistics. Data collection and processing.

Space Group	P 6_3_
Cell Parameters	
a, b, c (Å )	138.53, 138.53, 69.77
α, β, γ (°)	90, 90, 120
Wavelength (Å)	0.9184
Wilson B factor (Å^2^)	35.85
Resolution range (Å)	26.179–1.800 (1.864–1.800)
Completeness (%)	100.0 (100.0)
R_merge_ (%)	14.7 (205.2)
R_pim_ (%)	3.3 (46.0)
Observed reflections	1,461,081 (213,187)
Unique reflections	70,756 (10,268)
I/sigma (I)	14.57 (1.8)
Average multiplicity	20.6 (20.8)
Refinement	
Resolution (Å)	1.80
No. of reflections used	70,689
R_factor_ (%)	17.02
R_free_ (%)	19.65
Average B factor (Å^2^)	
Protein	31.75
Water	38.87
RMSD from Ideal Values	
Bond length (Å)	0.011
Bond angles (°)	1.088
Ramachandran Statistics (%)	
Most favored regions	97.10
Additionally allowed regions	2.90
Content of the Asymmetric Unit	
No. of protein molecules/residues/non-H atoms	4/556/8135
No. of solvent molecules	462

Data for the highest-resolution shell are provided in parentheses. RMSD, root-mean-square deviation.

**Table 2:** Results from DALI search with 6FMG structure.

PDB ID	Z-Scores	RMSD Å	Sequence Identity %
4TKZ	21.6	1.5	34
3IPR	20.9	1.6	30
1PDO	20.8	1.5	36
3LFH	19.9	1.6	30
3BED	19.0	1.7	26
5T3U	18.2	2.1	29
3MTQ	17.4	2.2	30
